# Cold-water, Sulphur and ‘the itch’: James Henry’s principles for conducting controlled trials (1843)

**DOI:** 10.1007/s11845-025-04027-x

**Published:** 2025-08-18

**Authors:** Max Cooper, Jo Middleton, Sarah Cooper

**Affiliations:** 1 Department of Primary Care and Public Health, Brighton and Sussex Medical School, Falmer, BN1 9PH UK; 2 Department of Neurology, Royal Sussex County Hospital, Eastern Road, Brighton, BN2 5BE UK

**Keywords:** Cold-water treatment, Controlled trial, Ireland, Itch, James Lind, Scabies

## Abstract

**Background:**

James Henry (1798–1876) was a Dublin physician and classical scholar best remembered for his translation of Virgil’s Aeneid. Little is recorded about his medical practice.

**Aim:**

To describe Henry's 1843 proposal for a controlled trial of treatment for ‘the itch’.

**Method:**

Qualitative examination of historical medical journals.

**Results:**

Henry suggests taking twelve patients and treating six with cold-water treatment and six with standard treatment (sulphur). Henry’s proposal sets out four general principles for selecting diseases to study the alleged benefits of cold-water treatment. First, the condition should be one upon which it is safe to experiment. Second, the disease should be ‘visible and tangible, and… cannot be simulated or misrepresented, or misunderstood’. Third, it should have no spontaneous recovery. Finally, there should already be a ‘certain [i.e. proven] remedy’. Henry had published in 1834 on sulphur as a laxative. By calling for twelve participants, it is possible that Henry’s proposal was influenced by the writing of Francis Hauksbee the Younger (1743) and James Lind (1753).

**Conclusion:**

Although there is no evidence that Henry’s trial ever took place, his proposal and principles for selecting a disease to study reveal a critical and ethical mind. It also constitutes a notable landmark on the journey towards formal controlled trials.

## Introduction

James Henry (1798–1876) was a Dublin physician and classical scholar who is best remembered for his translation of Virgil’s Aeneid [1]. In 1826 Henry married Anne Jane Castlefin and they resided at 6 Fitzwilliam Square East, Dublin [1, 2]. Henry received his MD from Trinity College, Dublin, in 1832 and was elected a Fellow to the King and Queen's [later Royal] College of Physicians in Ireland in 1826 [1]. Little is recorded about Henry’s medical practice [1] and this article goes some way to addressing that gap by examining his 1843 'Proposed Test of the Cold-Water Cure' [2]. This was published in the Provincial Medical Journal where preceding editions include multiple cases of cold water treatment administered topically, using a soaked sheet, by drinking, immersion or injection. His proposal states:

'Let a given number of patients—say six or twelve—laboring under the same disease, be placed in any public hospital—let the cold-water treatment applied to one-half the number, and the ordinary medical treatment to the other half, and let the result be examined, and reported on’ [2]

Henry then presents a list of conditions that would be *inappropriate* for this trial. First, he excludes fever, measles and scarlatina because ‘recovery takes place in all these diseases... under every method of treatment’ [2]. It is of note that Henry also offers an ethical reason: ‘there would be a moral impropriety in making these severe and dangerous diseases the first subjects of an experiment that might, possibly, compromise life' [2]. He suggests avoiding ‘rheumatic, or gouty, or dyspeptic, or hypochondriac patients’ and offers two reasons: ‘because they also recover under a great variety of methods of treatment, and because [they] may be simulated, or recovery may be alleged to have taken place where it has not, or not to have taken place where it has' [2]

With some repetition, he then sets out four key principles for choosing a disease for the trial:

'A disease should, therefore, be selected which is, First, Of such a nature that an experiment may be made on it with safety to the patient. Secondly, Which is visible and tangible, and what cannot be simulated or misrepresented, or misunderstood. Thirdly, From which there is no spontaneous or natural recovery, and in which, therefore, if recovery takes place, it is fairly attributable to the remedies used; and Lastly, A disease for which medical art at present possesses a certain remedy' [2].

Henry then proceeds to propose the ‘psora, or the itch’ as a condition that ‘possesses all these requisites' [2]. He calls for ‘six itch patients’ to receive cold-water treatment and another six the ‘common medical treatment by sulphur' [2]. He suggests that a fortnight would be sufficient time for the trial. He then proposes that ‘further trials upon a similar plan may then be made on other diseases' [2]. Finally, Henry makes it clear that his purpose is to question the assertion that cold-water could cure all conditions:

'If the result of this first experiment [i.e. on ‘the itch’] be unfavourable [sic] to the system [of cold-water treatment], the statement of its propounders and partisans must be modified, so that instead of saying it cures all curable diseases, they must limit themselves to the statement that it cures all curable diseases except the simplest and most easily curable of all, the itch' [2].

## Discussion

Henry’s proposal is a novel contribution to the field of cold-water research. Henry’s submission appears to have been triggered by claims of cure by cold-water treatment published in preceding issues of the journal. This points to a suspicion that many patients were paying handsomely for doctors to treat psychological and self-resolving conditions. It is of particular note that he believed that cold-water therapy should be compared with standard treatment. This clearly suggests that he considered sulphur to be more effective.

It is surprising that Henry considered ‘the itch’ to be an ideal condition for his proposed trial. That is because in the nineteenth century it represented a ‘mixed bag’ of conditions, most notably scabies [3]. That he had this in mind is hinted at by his proposal for a comparison with sulphur, already at that time a long-established treatment for scabies [4] and one which continues to be routinely used today in low-income countries [5]. Henry appears to have been particularly interested in sulphur. This is evidenced in his 1834 publication in The Edinburgh Medical and Surgical Journal. There in Henry describes its laxative property in an article entitled: ‘An Account of an Improved mode of Administering Sulphate of Magnesia (Epsom Salt), Whereby It Is Rendered an Agreeable, Safe, and Efficacious Purgative, Applicable to Almost Every Case in Which a Purgative Is Required’ [6].

## Conclusion

Henry’s proposal for a prospective trial appears to be the first to consider treatments of skin disease, although there is no evidence it ever took place. His proposal builds upon studies like the 1795 publication of William Falconer MD FRS (1744–1824). Using hospital registers from Bath, England, Falconer produced basic comparative ratios of clinical outcomes (‘no better’, ‘better' ‘much better’ or ‘cured) following water treatment [7]. It is possible that Henry was influenced by two predecessors in the field of controlled trials: the English instrument maker Francis Hauksbee the Younger (1688 − 1763) and the Scottish Royal Navy surgeon James Lind (1716–1794) [8]. That is because they both considered twelve patients to be a suitable participant size for a controlled trail. Like Henry, Hauksbee’s ‘plan’ suggests initially dividing them into two groups of six [8]. Henry’s proposal and principles of selecting a disease for a treatment trial reveal a critical and ethical mind. It also constitutes a notable landmark on the journey towards formal controlled trials.
